# Development and external validation of a diagnostic model for cardiometabolic-based chronic disease : results from the China health and retirement longitudinal study (CHARLS)

**DOI:** 10.1186/s12872-023-03418-1

**Published:** 2023-08-23

**Authors:** Yong Li

**Affiliations:** Department of General Medicine, Beijing Anzhen Hospital, Capital Medical University, No. 2 Anzhen Road, Chaoyang District, Beijing, 100029 China

**Keywords:** Cardiometabolic diseases, Hypertension, Insulin resistance, Risk factors, Nomograms

## Abstract

**Background:**

Cardiovascular disease(CVD) is the leading cause of death in the world. Cardiometabolic-based chronic disease (CMBCD) model is presented that provides a basis for sustainable and early, evidence-based therapeutic targeting to mitigate the ravagest and development of CVD. CMBCD include dysglycemia, hypertension, and/or dyslipidemia progressing to downstream CVD events.

**Objectives:**

The objective of our research was to develop and externally validate a diagnostic model of CMBCD.

**Methods:**

Design: Multivariable logistic regression of a cohort for 9,463 participants aged at least 45 years were drawn from the 2018 wave of the China Health and Retirement Longitudinal Study (CHARLS). Setting: The 2018 wave of the CHARLS. Participants:Diagnostic model development: Totally 6,218 participants whose individual ID < 250,000,000,000. External validation: Totally 3,245 participants whose individual ID > 250,000,000,000. Outcomes: CMBCD .

**Results:**

CMBCD occurred in 25.5%(1,584/6,218)of individuals in the development data set and 26.2%(850 /3,245)of individuals in the validation data set. The strongest predictors of CMBCD were age, general health status, location of residential address, smoking, housework ability, pain, and exercise tolerance. We developed a diagnostic model of CMBCD. Discrimination was the ability of the diagnostic model to differentiate between people who with and without CMBCD. This measure was quantified by calculating the area under the receiver operating characteristic(ROC) curve(AUC).The AUC was 0.6199 ± 0.0083, 95% confidence interval(CI) = 0.60372 ~ 0.63612. We constructed a nomograms using the development database based on age, general health status, location of residential address, smoking, housework ability, pain, and exercise tolerance. The AUC was 0.6033 ± 0.0116, 95% CI = 0.58066 ~ 0.62603 in the validation data set.

**Conclusions:**

We developed and externally validated a diagnostic model of CMBCD. Discrimination, calibration, and decision curve analysis were satisfactory.

**Supplementary Information:**

The online version contains supplementary material available at 10.1186/s12872-023-03418-1.

## Introduction

Cardiovascular disease (CVD) is the leading cause of death in the world [1–3]. CVD includes a wide spectrum of disorders that affect the heart and blood vessels [1]. In many individuals, the treatment of CVD begins with the onset of events . [1]Although overt CVD are the domain of adulthood, it is evident that the CVD continuum begins very early in life [4]. Recognition of risk factors and early stages of CVD damage, at a time when these processes are still reversible, and the development of prevention strategies are major pillars in reducing CVD morbidity and mortality in the general population [5]. Cardiometabolic-based chronic disease (CMBCD) model is presented that provides a basis for early and sustainable, evidence-based therapeutic targeting to mitigate the ravagest and development of CVD. The phenomenon of derangement of metabolic inflexibility is a common thread linking insulin resistance to CMBCD [4]. CMBCD include hypertension, dysglycemia, and/or dyslipidemia progressing to downstream CVD events [6, 7]. The 2 upstream metabolic drivers of CMBCD are adiposity and dysglycemia [4]. These metabolic drivers interact at the level of insulin resistance, and have been previously configured as adiposity-based chronic disease (ABCD) and dysglycemia-based chronic disease (DBCD) [8, 9].

There are 4 CMBCD stages: risk development, pre-disease, disease, and complications [4] . Clinicians should approach individuals using the CMBCD model to incorporate lifestyle changes as early as possible to optimally mitigate the burden of CMBCD.

Primary care physicians engaged in preventive health maintenance want to assess risk of developing any CMBCD event using a general CMBCD risk assessment tool [10]. There exists a need for tools that will be able to aid early identification of individuals at increased risk of CMBCD. We want to develop and externally validate a diagnostic model of CMBCD. The aim of our study was 4-fold: (1) to identify predictive factors; (2) to develop a diagnostic model; (3) to create a nomogram and (4) to externally validate diagnostic model.

## Methods

We followed the methods of Li Y. 2020 [11].

Data were from the 2018 wave of the China Health and Retirement Longitudinal Study (CHARLS), a nationally representative longitudinal survey of people aged 45 years old or above in China [12]CHALRS collects high-quality multidisciplinary data, including basic demographics, health information, and socioeconomic status . [12]. We used type 2b of prediction model studies covered by Transparent Reporting of a multivariable prediction model for Individual Prognosis Or Diagnosis (TRIPOD) statement [13]. The data were nonrandomly split by ID number into 2 groups: one to develop the prediction model and one to evaluate its predictive performance [13]. Type 2b was referred to as “external validation studies” [13]. The derivation cohort was 6,218 CMBCD whose individual ID < 250,000,000,000. The validation cohort was 3,245 CMBCD whose individual ID > 250,000,000,000.

In CHARLS, the presence of chronic disease was assessed by the question, “Have you been diagnosed by a doctor with the following conditions?“ [12]. There were 14 chronic conditions in total, including hypertension, dyslipidemia, diabetes, cancer, chronic lung diseases, chronic liver disease, heart problems (i.e., heart attack, coronary heart disease, angina, congestive heart failure), stroke, and so on [12]. Each condition was coded as a dichotomous variable, with the presence of disease = 1 [12]. Obesity and type 2 diabetes are major cardiometabolic drivers, represented as distinct stages of ABCD and DBCD, respectively, and leading to CMBCD [14]. CMBCD was defined as the presence of at least one of the heart disease, stroke, diabetes, hypertension, and dyslipidemia in a single individual.

Inclusion criteria: defined as CMBCD and age of more than or equal 45 years.

Exclusion criteria: age of less than 45 years.

Outcome of interest was CMBCD. The absence or presence of CMBCD was decided blinded to the predictor variables and based on the survey record [11, 13]. 

We selected 12 predictor according to the results of baseline descriptive statistics and clinical relevance [11]. The potential candidate variables were biological (age, sex, pain, sleep duration, and general health status) and social (housework ability, smoking, alcohol consumption, location of residential address, exercise tolerance, marital status, and education level) determinants of health. All of them based on the survey record. In regression analysis, a dummy variable is a regressor that can take only two values: either 1 or 0.Dummy variables are typically used to encode categorical features. Smoking was defined as a dummy variable, which equals 1 if an individual was a current or past smoker, and 0 if an individual has never smoked [12]. Alcohol consumption was also defined as a dummy variable (never vs. past or current) [12]. Location of residential address was defined as a dummy variable, which equals 1 if an individual live in the central of city/town, and 0 if an individual live in rural or urban-rural integration zone [12]. Marital status was defined as a dummy variable, which equals 1 if an individual married with spouse present, and 0 if an individual married but not living with spouse temporarily for reasons such as work, separated, divorced, widowed, and never married [12]. Sleep duration was defined during last month average hours of actual sleep [12]. Exercise tolerance was defined as a dummy variable, which equals 1 if an individual don’t have any difficulty or have difficulty but can still run or jog about 1Km, and 0 if an individual have difficulty and need help or can not run or jog about 1Km [12]. Education level was defined as a dummy variable, which equals 1 if an individual finished primary school, and 0 if an individual did not finish primary school. [12] General health status was defined as a dummy variable,which equals 1 if an individual said his health was poor or very poor, and 0 if an individual said his health was very good, good, or fair [12]. Pain was defined as a dummy variable,which equals 1 if an individual said any part of his body feel pain, and 0 if an individual said no part of his body feel pain [12]. Housework ability was defined as a dummy variable,which equals 1 if an individual said he did not have any problem to do housework, and 0 if an individual said he was unable to do housework or he could not do housework for an extended period of time [12].

Our sample and the number of events exceed all approaches for determining samples sizes and therefore are expected to provide estimates that are very robust [11, 13]. To ensure reliability of data, we excluded individuals who had missing information on key predictors: age, general health status, housework ability, pain, location of residential address, smoking, and exercise tolerance [11, 13].

We used univariable and multivariable logistic regression models to identify the correlates of CMBCD [11, 13]. We entered all variables of Table 1 into the univariable logistic regression [11]. We constructed a multivariable logistic regression model using the backward variable selection method, based on the variables that resulted significant from univariable logistic regression [11]. We used the Bayesian information criterion (BIC) and Akanke information criterion (AIC)to select predictors [11]. It accounts for model fit while penalizing for the number of parameters being estimated and corresponds to using α = 0.157. [11, 13]

We assessed the predictive performance of the diagnostic model in the validation data sets by examining measures of discrimination, calibration, and decision curve analysis (DCA) [11, 13].

We performed statistical analyses with STATA version 15.1, R version 4.2.1 and the RMS package developed by Harrell [11].

## Results

Totally 25.5%(1,584/6,218)individuals suffered CMBCD in the development data set. Baseline characteristics of the individuals were shown in Table 1. Nine variables (age, general health status, location of residential address, smoking, alcohol consumption, housework ability, pain, sleep duration, and exercise tolerance)were significant differences in the two groups of individuals( p < 0. 157). After application of backward variable selection method, AIC, and BIC, age, general health status, location of residential address, smoking, housework ability, pain, and exercise tolerance remained as significant independent predictors of CMBCD [11]. Results were shown in Tables 2 and 3.

**Table 1:** Demographic and clinical characteristics of individuals with and without CMBCD in the development data sets

Characteristic [lower limit, upper limit]	Total (n = 6,218)	CMBCD (n = 1,584)	No CMBCD (n = 4,634)	P value
Age (year, x ± s) [45,98]	60 ± 9	61 ± 9	60 ± 9	< 0.001
Man n ( %) 0 = No, 1 = Yes	3,113 (50.1)	813(51.3)	2,300(49.6)	0.245
Smoking n ( %) 0 = No, 1 = Yes	2,709(43.6)	715(45.1)	1,994 (43.0)	0.144
Alcohol consumption n ( %)0 = No, 1 = Yes	2,343(37.7)	561(35.4)	1,782(38.5)	0.031
Location of residential address n ( %) 0 = No,1 = Yes	1,088(17.5)	338(21.3)	750 (16.2)	< 0.001
Education level n ( %) 0 = No,1 = Yes	4,156 (66.9)	1,104(69.7)	3,052(65.9)	0.267
Marital status n ( %)0 = No, 1 = Yes	4,998(80.4)	1,256(79.3)	3,742(80.8)	0.207
Sleep duratio n(hour, x ± s) [0,20]	6.3 ± 1.8	6.2 ± 1.9	6.4 ± 1.8	< 0.001
Exercise tolerance n (%)0 = No, 1 = Yes	4,355(70.0)	993 (62.7)	3,362(72.6)	< 0.001
Housework ability n (%)0 = No, 1 = Yes	5,234(84.2)	1,229 (77.6)	4,005(86.4)	< 0.001
General health status n (%)0 = No, 1 = Yes	1,074(17.3)	411 (25.9)	663(14.3)	< 0.001
Pain n (%)0 = No, 1 = Yes	1,403(22.6)	478 (30.2)	925(20.0)	< 0.001

**Table 2:** Predictor of CMBCD obtained from multivariable logistic regression models(odds ratio)in the development data set

CMBCD	Odds ratio	Std.Err	Z	P>| Z |	95% CI
Age	1.010093	0.0034962	2.90	0.004	1.003264 ~ 1.016969
Location of residential address	1.577025	0.1184411	6.07	<0.001	1.361162 ~ 1.827121
Smoking	1.203021	0.0735907	3.02	0.003	1.067097 ~ 1.356259
Exercise tolerance	0.828159	0.058211	-2.68	0.007	0.7215776 ~ 0.950483
Housework ability	0.7484253	0.0614902	-3.53	< 0.001	0.6371096 ~ 0.8791899
General health status	1.685621	0.1325399	6.64	< 0.001	1.444875 ~ 1.96648
Pain	1.524438	0.1078588	5.96	< 0.001	1.327042 ~ 1.751197
_cons	0.1855257	0.045599	-6.85	< 0.001	0.1146024 ~ 0.3003409

CI = confidence interval.

**Table 3:** Predictor of CMBCD obtained from multivariable logistic regression models(Coef) in the development data sets

CMBCD	Coef	Std.Err	Z	P>| Z |	95% CI
Age	0.0100423	0.0034613	2.90	0.004	0.0032583 ~ 0.0168263
Location of residential address	0.4555399	0.0751041	6.07	<0.001	0.3083385 ~ 0.6027413
Smoking	0.1848358	0.0611716	3.02	0.003	0.0649418 ~ 0.3047299
Exercise tolerance	− 0.1885502	0.0702897	-2.68	0.007	− 0.3263154~-0.050785
Housework ability	− 0.2897839	0.0821595	-3.53	< 0.001	− 0.4508135~-0.1287543
General health status	0.5221339	0.0786297	6.64	< 0.001	0.3680225 ~ 0.6762454
Pain	0.4216258	0.0707531	5.96	< 0.001	0.2829522 ~ 0.5602994
_cons	-1.684562	0.2457824	-6.85	< 0.001	-2.166286 ~ -1.202837

CI = confidence interval.

**Fig. 1:**
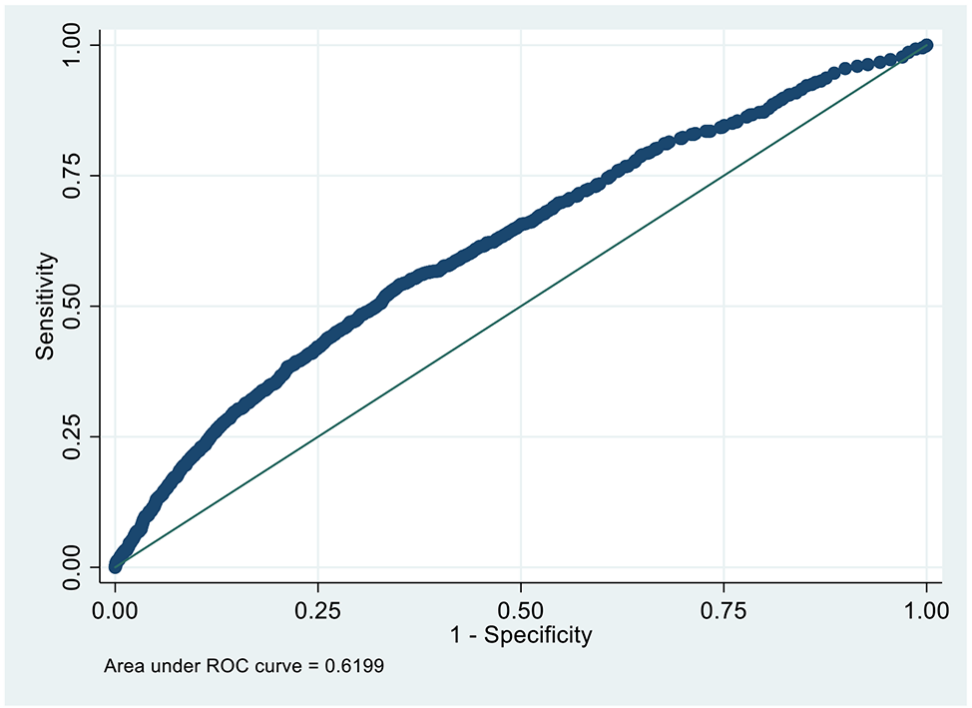
Receiver-operating characteristics curve in identifying individuals with CMBCD in the development dataset

According to the above risk factors, we can calculate the predicted probability of CMBCD using the following formula: P = 1/(1 + exp(-( -1.684562 + − 0.1885502 *ET + 0.0100423 *AGE(year) + 0.4555399*ADDRESS + 0.5221339*HA + 0.1848358*SMOKE + 0.5221339*GHS + 0.4216258*PAIN))) [11]. AGE = age(year), ET = exercise tolerance(0 = No, 1 = Yes), ADDRESS = location of residential address (0 = No, 1 = Yes), SMOKE = smoking (0 = No, 1 = Yes), HA = housework ability (0 = No, 1 = Yes), GHS = general health status (0 = No, 1 = Yes), PAIN = pain (0 = No, 1 = Yes). We drew the ROC curve (Fig. 1). AUC was 0.6199 ± 0.0083, 95% confidence interval(CI) = 0.60372 ~ 0.63612.

**Fig. 2:**
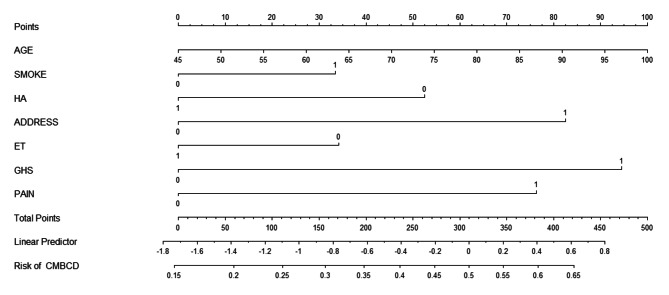
A nomograms for predicting CMBCD. AGE = age(year), ET = exercise tolerance(0 = No, 1 = Yes), ADDRESS = location of residential address (0 = No, 1 = Yes), SMOKE = smoking (0 = No, 1 = Yes), HA = housework ability (0 = No, 1 = Yes), GHS = general health status(0 = No, 1 = Yes), PAIN = pain (0 = No, 1 = Yes)

We constructed the nomograms (Fig. 2) using the development database based on seven independent prognostic marker : age, housework ability, pain, location of residential address, smoking, general health status, and exercise tolerance [11].

**Fig. 3:**
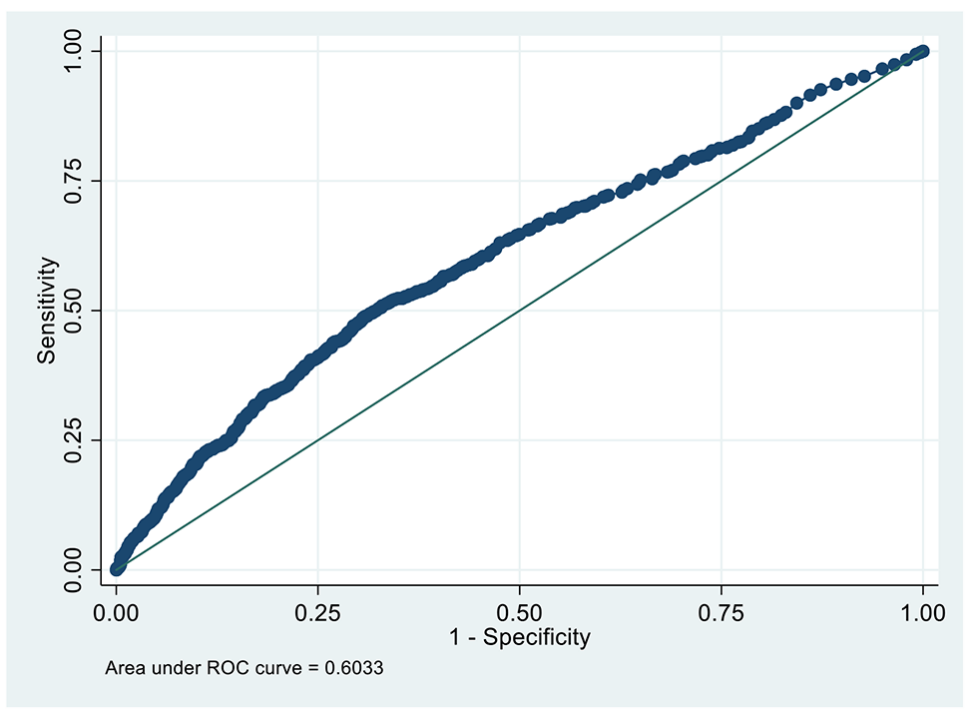
Receiver-operating characteristics curve in identifying individuals with CMBCD in the validation data sets

Totally 26.2%(850 /3,245)individuals suffered CMBCD in the validation data sets. Baseline characteristics of the individuals were shown in Table 4.We can calculate the predicted probability of CMBCD using the following formula: P = 1/(1 + exp(-( -1.684562 + − 0.1885502 *ET + 0.0100423 *AGE(year) + 0.4555399*ADDRESS + 0.5221339*HA + 0.1848358*SMOKE + 0.5221339*GHS + 0.4216258*PAIN))) [11]. AGE = age(year), ET = exercise tolerance (0 = No, 1 = Yes), ADDRESS = location of residential address (0 = No, 1 = Yes), SMOKE = smoking (0 = No, 1 = Yes), HA = housework ability (0 = No, 1 = Yes), GHS = general health status (0 = No, 1 = Yes), PAIN = pain (0 = No, 1 = Yes). We drew the ROC curve (Figure 3). AUC was 0.6033 ± 0.0116, 95% CI = 0.58066 ~ 0.62603.

**Table 4:** Demographic and clinical characteristics of individuals with and without CMBCD in the validation data sets

Characteristic [lower limit, upper limit]	Total (n = 3,245)	CMBCD (n = 850)	No CMBCD (n = 2,395)	P value
Age (year, x ± s) [45,89]	59 ± 9	60 ± 9	58 ± 8	< 0.001
Man n ( %) 0 = No, 1 = Yes	1,623 (50.0)	405(47.6)	1,218(50.9)	0.108
Smoking n ( %) 0 = No, 1 = Yes	1,335(41.1)	338(39.8)	997 (41.6)	0.343
Alcohol consumption n ( %)0 = No, 1 = Yes	1,265(39.0)	316(37.2)	949(39.6)	0.209
Location of residential address n ( %) 0 = No,1 = Yes	464(14.3)	147(17.3)	317 (13.2)	0.004
Education level n ( %) 0 = No,1 = Yes	2,184 (67.3)	587(69.1)	1,597(66.7)	0.638
Marital status n ( %)0 = No, 1 = Yes	2,692(83)	701(82.5)	1,991(83.1)	0.660
Sleep duration (hour, x ± s) [0,20]	6.3 ± 1.9	6.2 ± 1.8	6.3 ± 1.9	0.033
Exercise tolerance n (%)0 = No, 1 = Yes	2,340(72.1)	550 (64.7)	1,790(74.7)	< 0.001
Housework ability n (%)0 = No, 1 = Yes	2,794(86.1)	697 (81.6)	2,097(87.6)	< 0.001
General health status n (%)0 = No, 1 = Yes	512(15.8)	212 (24.9)	300(12.5)	< 0.001
Pain n (%)0 = No, 1 = Yes	600(18.5)	193 (22.7)	407(17.0)	< 0.001

**Fig. 4:**
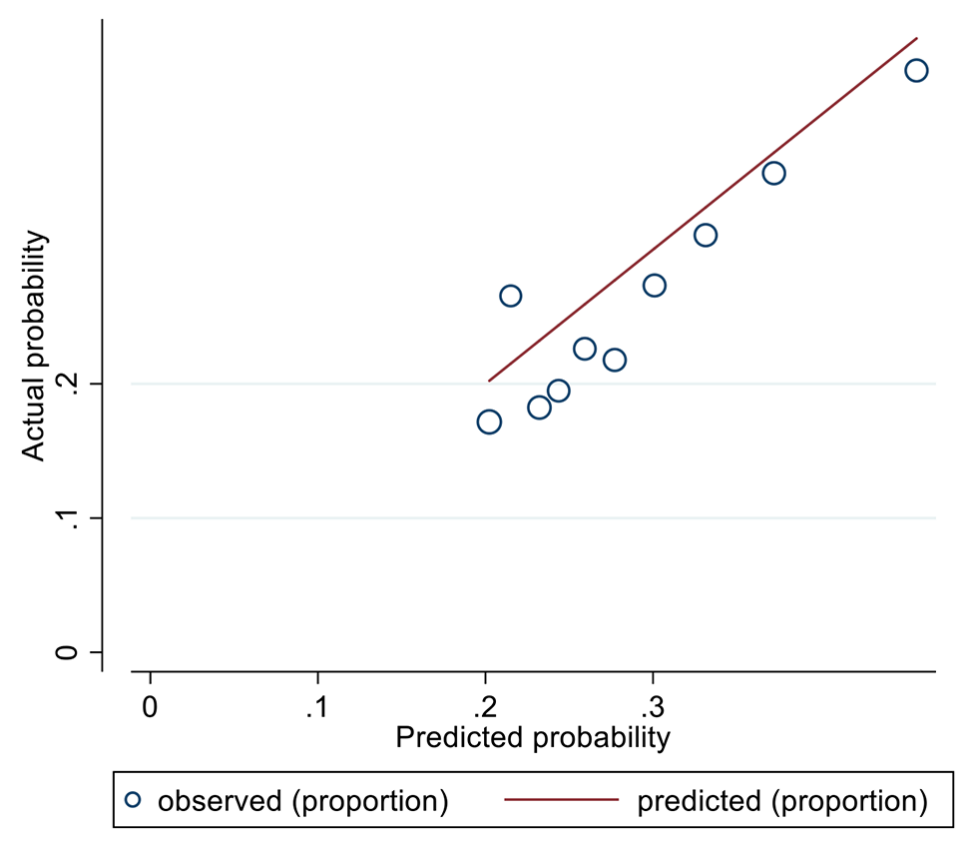
A calibration plot with distribution of the predicted probabilities for individuals with and without CMBCD in the validation data sets

We drew a calibration plot (Fig. 4) with distribution of the predicted probabilities for individuals with and without CMBCD in the validation data sets [11]. Hosmer-Lemeshow chi2(10) = 25.69, Prob > chi2 = 0.0042. Brier score = 0.1159 < 0.25.

**Fig. 5:**
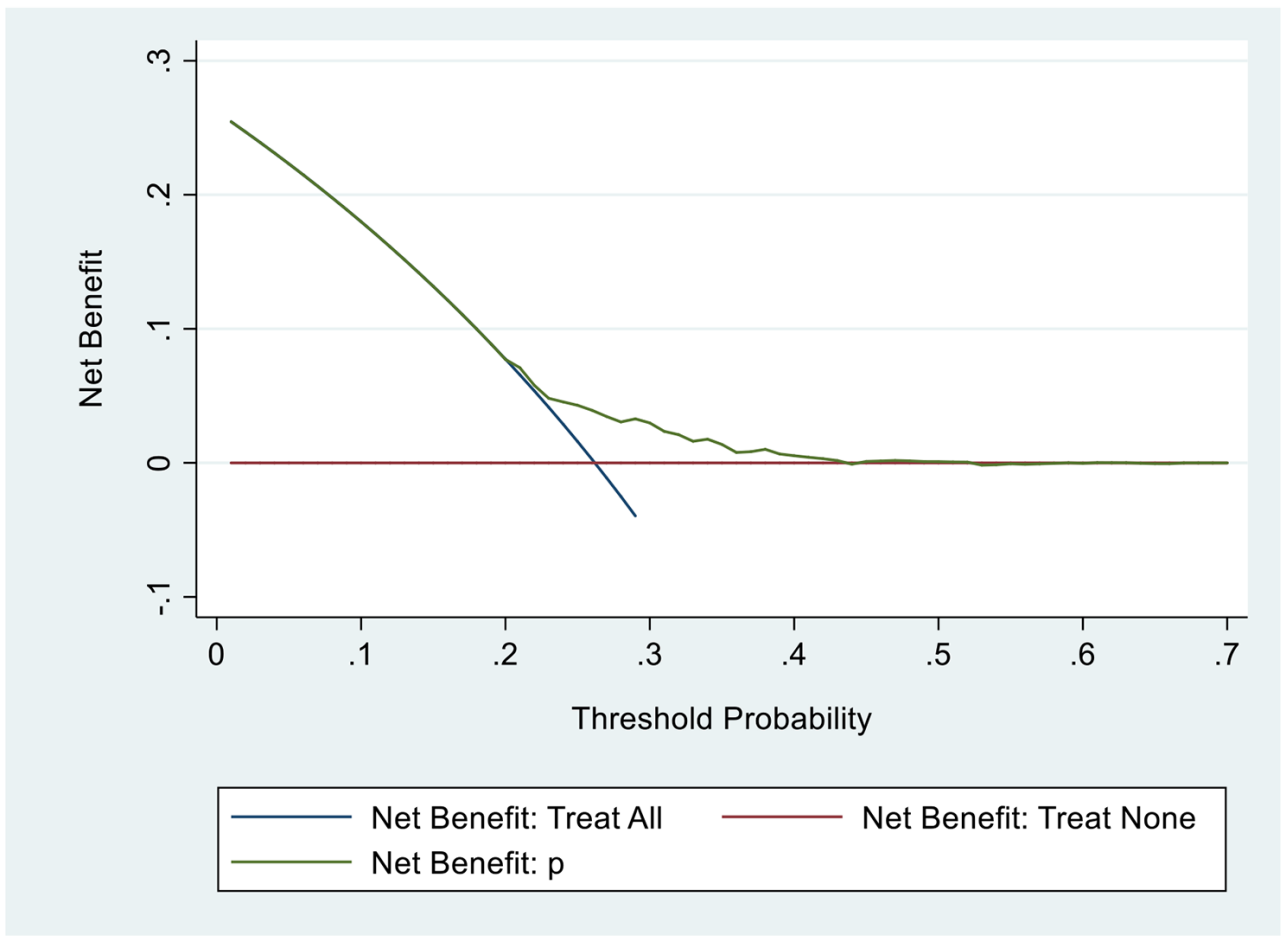
DCA in the validation data sets

DCA(Fig. 5) in the validation data sets. [11, 13]

## Discussion

We assessed the predictive performance of the diagnostic model in the validation data sets by examining measures of discrimination, calibration, and DCA [11, 13]. Discrimination, calibration, and DCA were satisfactory. In our study, age, general health status, location of residential address, smoking, housework ability, pain, and exercise tolerance are associated with an increased risk of CMBCD. We can use nomograms or the formula to predict CMBCD [11]. We can use specific strategies to reduce CMBCD risk such as quiting smoking.

The Framingham Heart Study is a sex-specific multivariable risk factor algorithm can be conveniently used to assess general CVD risk and risk of individual CVD events [10]. The estimated absolute CVD event rates can be used to quantify risk and to guide preventive care [10]. World Health Organization cardiovascular disease risk charts estimate 10-year predicted risk in 21 global regions [15]. The JBS3 risk score predicts both a short-term risk (10-year) and the lifetime risk of CVD using conventional and non-conventional risk factors [16]. The JBS3 risk score recognizes and encompasses a large patient population at a lower or intermediate 10-year risk but has a high lifetime risk [16]. The Globorisk cardiovascular risk equation can be recalibrated and updated for use in difffferent countries with routinely available information [17].

Any numerical summary derived from a risk calculator is not the risk of an individual [16]. Population based estimates for risk factor modification effects on CVD outcomes are not extrapolated for use in an individual [16]. These estimates only provide the individual with a reasonable guide to the potential benefits of risk modification [16]. Both the prevalence and the incidence of CMBCD can help us get multiple risk factors.The prevalence of CMBCD is easy get than the incidence of CMBCD does. For these reasons, we can use the prevalence of CMBCD replace the incidence of CMBCD.

Advanced age has been reported to be an independent risk factor of CMBCD [18]. Chronological aging might contribute to atherosclerosis [18]. Aging and inflammation both contribute to CVD [3]. The center between metabolic diseases, inflammation, aging, and cardiovascular is microcirculation [19], There was a strong link between vascular risk factors, somatic hematopoietic mutations, and age-related CVD [20].

In our study, individuals who said any part of his body feel pain had a 1.52 risk of developing CMBCD compared with individuals who said no part of his body feel pain. Pain can be divided into two main types: acute and chronic pain [21]. Chronic pain affects a large part of the population causing functional disability, being often associated with coexisting psychological disorders [21]. Aging has been linked to a decrease in pain tolerance, a decline of painful sensations, and an increase in the pain threshold [21]. The prevalence and incidence of CMBCD increases as a function of age [18].

Exercise tolerance and housework ability was associated with a lower risk of CMBCD. In our study, individuals who don’t have any difficulty or have difficulty but can still run or jog about 1Km had a 0.83 risk of developing CMBCD compared with individuals who have difficulty and need help or can not run or jog about 1Km. Individuals who don’t have any problem to do housework had a 0.75 risk of developing CMBCD compared with individuals who was unable to do housework or could not do housework for an extended period of time.

Smoking has been reported to be an independent risk factor of CMBCD [22–24]. In our study, individuals who smoked had a 1.20 risk of developing CMBCD compared with individuals who did not smoke. Nicotine increases insulin resistance [23, 24]. Smoking obstruct the function of endocrine system [22]. Tobacco smoking impairs the regular metabolic pathway [22]. Smoking play a role in the development of somatic mutations [20]. Smoking has been associated with the bias explaining the ‘obesity paradox’ [23, 25]. 

Urban rural differences is associated with the risk of CMBCD. In our study, urban individuals had a 1.58 risk of developing CMBCD compared with rural or urban-rural individuals. The prevalence of CMBCD was greater in rural than that in urban areas in Korea [26]. Among Kenyan, 9.38% of the women were hypertensive with higher prevalence among urban 11.61%, compared to rural women, 7.86% [27]. The prevalence of diabetes was lower among respondents living in rural areas [prevalence odds ratio (POR) = 0.94, P = 0.032], but the prevalence of coronary heart disease was higher (POR = 1.09, P = 0.011) [28]. The likelihood of new onset T2DM by community type varied by region of the United States [29].

The strengths of this study include several ways. It includes only baseline factors, including age, general health status, location of residential address, smoking, housework ability, pain, and exercise tolerance. It is easily calculated at patient presentation [11]. It is not a relative value but an absolute value [11]. The nomograms we constructed for CMBCD captures the majority of diagnostic information offered by a full logistic regression model [11].

Our study has several important limitations. First, all CMBCD conditions and most of the variables come from respondent self reporting, which may cause potential bias. Second, other potentially influential factors such as obesity were not involved due to the limitations of the secondary data set. Finally, the c statistic of the study CMBCD model at 0.6199 in the derivation and 0.6033 in the validation cohort is modest. A formal comparison of the score on a second cohort could prove very useful. Given the mostly yellow CHARLS sample, the transportability of the CMBCD risk function in other samples must be evaluated.

In our study, age, general health status, location of residential address, smoking, housework ability, pain, and exercise tolerance are associated with an increased risk of CMBCD.These estimates emphasise the importance of early intervention of multiple risk factors [16].

## Conclusion

We developed and externally validated a moderate diagnostic model of CMBCD. Discrimination, calibration, and DCA were satisfactory.

### Electronic supplementary material

Below is the link to the electronic supplementary material.

Additional File 1: Checklist of items to include when reporting a study developing or validating a multivariable prediction model for diagnosis or prognosis*

## Data Availability

The data used in this study are released data by CHARLS for public use. Permissions were acquired to access the data used in our research, which were granted by CHARLS team. The raw data is available on website (http://charls.pku.edu.cn/en).

## Abbreviations

AIC: Akanke information criterion
AUC: area under the receiver operating characteristic curve
BIC: Bayesian information criterion
CHARLS: The China Health and Retirement Longitudinal Study
CI: confidence interval
CMBCD: Cardiometabolic-based chronic disease
CVD: Cardiovascular Diseases
ROC: receiver operating characteristic
TRIPOD: Transparent Reporting of a multivariable prediction model for Individual Prognosis Or Diagnosis
