# Supplementary material for: Development and external validation of a diagnostic model for cardiometabolic-based chronic disease : results from the China health and retirement longitudinal study (CHARLS)
Source: BMC Cardiovasc Disord. 2023 Aug 23;23:417. doi: 10.1186/s12872-023-03418-1 (PMC10464030; doi:10.1186/s12872-023-03418-1)
Supplement: Supplementary file 1 — Additional File 1: Checklist of items to include when reporting a study developing or validating a multivariable prediction model for diagnosis or prognosis* [file 12872_2023_3418_MOESM1_ESM.docx]

BMJ 2014;350:g7594 doi: 10.1136/bmj.g7594 (Published 7 January 2015) Page 6 of 9

RESEARCH METHODS & REPORTING

**Table**

**Supplementary table 1. Checklist of items to include when reporting a study developing or validating a multivariable prediction model for diagnosis or prognosis***

| **Section/topic** | **Item** | **Development or**  **validation?** | **Checklist item Page** |  |
| --- | --- | --- | --- | --- |
| **Title and abstract** | | | |  |
| Title | 1 | D;V | Identify the study as developing and/or validating a multivariable prediction model, the target population, and the outcome to be predicted | 1 |
| Abstract | 2 | D;V | Provide a summary of objectives, study design, setting, participants, sample size, predictors, outcome, statistical analysis, results, and conclusions | 1,2 |
| **Introduction** | | | | **2** |
| Background and  objectives | 3a | D;V | Explain the medical context (including whether diagnostic or prognostic) and rationale for developing or validating the multivariable prediction model, including references to existing models | 2 |
|  | 3b | D;V | Specify the objectives, including whether the study describes the development or validation of the model, or both | 2 |
| **Methods** | | | | **3** |
| Source of data | 4a | D;V | Describe the study design or source of data (for example, randomised trial, cohort, or registry data), separately for the development and validation datasets, if applicable | 3 |
|  | 4b | D;V | Specify the key study dates, including start of accrual; end of accrual; and, if applicable, end of follow-up | 3 |
| Participants | 5a | D;V | Specify key elements of the study setting (for example, primary care, secondary care, general population) including number and location of centres | 3 |
|  | 5b | D;V | Describe eligibility criteria for participants | 4 |
|  | 5c | D;V | Give details of treatments received, if relevant | No treatments |
| Outcome | 6a | D;V | Clearly define the outcome that is predicted by the prediction model, including how and when assessed | 4 |
|  | 6b | D;V | Report any actions to blind assessment of the outcome to be predicted | 4 |
| Predictors | 7a | D;V | Clearly define all predictors used in developing the multivariable prediction model, including how and when they were measured | 4，5 |
|  | 7b | D;V | Report any actions to blind assessment of predictors for the outcome and other predictors | no |
| Sample size | 8 | D;V | Explain how the study size was arrived at. | 5 |
| Missing data | 9 | D;V | Describe how missing data were handled (for example, complete-case analysis, single imputation, multiple imputation) with details of any imputation method | 5 |
| Statistical analysis  methods | 10a | D | Describe how predictors were handled in the analyses | 5 |
|  | 10b | D | Specify type of model, all model-building procedures (including any predictorselection), and method for internal validation | 5 |
|  | 10c | V | For validation, describe how the predictions were calculated | 5 |
|  | 10d | D;V | Specify all measures used to assess model performance and, if relevant, to compare multiple models | 5 |
|  | 10e | V | Describe any model updating (for example, recalibration) arising from the validation, if done | No model updating |
| Risk groups | 11 | D;V | Provide details on how risk groups were created, if done | No risk groups |
| Development v  validation | 12 | V | For validation, identify any differences from the development data insetting, eligibility criteria, outcome, and predictors | 3 |
| **Results** | | | |  |
| Participants | 13a | D;V | Describe the flow of participants through the study, including the number of participants with and without the outcome and, if applicable, a summary of the follow-up time. A diagram may be helpful | 5 |
|  | 13b | D;V | Describe the characteristics of the participants (basic demographics, clinical features, available predictors), including the number of participants with missing data for  predictors and outcome | 5 |
|  | 13c | V | For validation, show a comparison with the development data of the distribution of important variables (demographics, predictors and outcome). | 10 |

Model development 14a D Specify the number of participants and outcome events in each analysis 6

| For personal use only: See rights and reprints <http://www.bmj.com/permissions> Subscribe: <http://www.bmj.com/subscribe> |
| --- |

BMJ 2014;350:g7594 doi: 10.1136/bmj.g7594 (Published 7 January 2015) Page 7 of 9

RESEARCH METHODS & REPORTING

**(continued)**

| **Section/topic** | **Item** | **Development or**  **validation?** | **Checklist item Page** |  |
| --- | --- | --- | --- | --- |
|  | 14b | D | If done, report the unadjusted association between each candidate predictor and outcome | 6 |
| Model specification | 15a | D | Present the full prediction model to allow predictions for individuals (that is, all  regression coefficients, and model intercept or baseline survival at a given time point) | 7，8 |
|  | 15b | D | Explain how to use the prediction model | 9 |
| Model performance | 16 | D;V | Report performance measures (with CIs) for the prediction model | 9，10，11，12 |
| Model updating | 17 | V | If done, report the results from any model updating (that is, model specification, model performance) | No model updating |
| **Discussion** | | | | 13 |
| Limitations | 18 | D;V | Discuss any limitations of the study (such as nonrepresentative sample, few events per predictor, missing data) | 15 |
| Interpretation | 19a | V | For validation, discuss the results with reference to performance in the development data, and any other validation data | no |
|  | 19b | D;V | Give an overall interpretation of the results, considering objectives, limitations, results from similar studies, and other relevant evidence | 16 |
| Implications | 20 | D;V | Discuss the potential clinical use of the model and implications for future research | 13 |
| **Other information** | | | |  |
| Supplementary  information | 21 | D;V | Provide information about the availability of supplementary resources, such as study protocol, Web calculator, and data sets | 17 |
| Funding | 22 | D;V | Give the source of funding and the role of the funders for the present study | 17 |
| *Items relevant only to the development of a prediction model are denoted by D, items relating solely to a validation of a prediction model are denoted by V, and items relating to both are denoted D;V. We recommend using the TRIPOD Checklist in conjunction with the TRIPOD explanation and elaboration document. | | | |  |

| For personal use only: See rights and reprints <http://www.bmj.com/permissions> Subscribe: <http://www.bmj.com/subscribe> |
| --- |
